# Supplementary material for: MetaRibo-Seq measures translation in microbiomes
Source: Nat Commun. 2020 Jun 29;11:3268. doi: 10.1038/s41467-020-17081-z (PMC7324362; doi:10.1038/s41467-020-17081-z)
Supplement: Supplementary file 10 — Supplementary Data 7 [file 41467_2020_17081_MOESM10_ESM.zip › File2/Confidence_VeryHigh_Taxonomy/5449_out.krona.html]

Javascript must be enabled to view this page.

members
magnitude
magnitudeUnassigned
count
unassigned
taxon
rank

5449\_out

7

2
7
superkingdom

1239
7
phylum

class
186801
7

2
7

SRS015431\_contig\_number\_65414SRS148159\_contig\_number\_52927
order
186802

186803
2
family

genus
1432051
2

1720294
2
species

SRS014459\_contig\_number\_contig-100\_27897.27898SRS143895\_contig\_number\_6318

3
216572
family

3
459786
genus

1
876091

SRS147614\_contig\_number\_contig-100\_4862.73073
species

2
1897011
species

SRS050752\_contig\_number\_5344SRS050941\_contig\_number\_11897
